# Flesh-Colored Papules on the Glans Penis

**DOI:** 10.3390/dermatopathology13020026

**Published:** 2026-06-10

**Authors:** Phatcharawat Chirasuthat, Supaporn Suwanchote, Tanaporn Borriboon

**Affiliations:** Department of Medical Services, Institute of Dermatology, Ministry of Public Health, Bangkok 10400, Thailand; bunji-taru@hotmail.com (P.C.); tanaporn.borriboon@gmail.com (T.B.)

**Keywords:** palisaded encapsulated neuroma, solitary circumscribed neuroma, peripheral nerve sheath tumor, spindle cell tumor, cutaneous neuroma, glans penis, immunohistochemistry

## Abstract

A 23-year-old man reported a 3-year history of slowly growing, slightly itchy, flesh-colored papules on the distal glans penis. He denied any history of trauma or previous treatment. Additionally, he experienced no difficulties with urination, discharge, or erectile function. Upon examination, three firm, dome-shaped papules were found to be attached to the skin but mobile over the underlying tissues. There was no regional lymph node enlargement, hardening, or fluctuation observed. Histopathological analysis revealed a well-defined, encapsulated tumor in the dermis, consisting of spindle cells interspersed with varying numbers of axons.

## Simple Summary

Flesh-colored papules on the glans penis represent a diagnostic challenge due to the wide range of possible etiologies. We describe a 23-year-old man with slowly growing, flesh-colored papules on the glans penis, present for three years without pain or urinary symptoms. Histopathological examination revealed a well-circumscribed dermal nodule of spindle cells arranged in interlacing fascicles with cleft formation, supported by immunohistochemical findings. This case highlights the importance of integrated histopathological and immunohistochemical evaluation in diagnosing unusual penile lesions, and demonstrates that complete surgical excision is both diagnostic and curative, with no known risk of recurrence or malignant transformation.

## 1. Case Presentation

**Figure 1:**
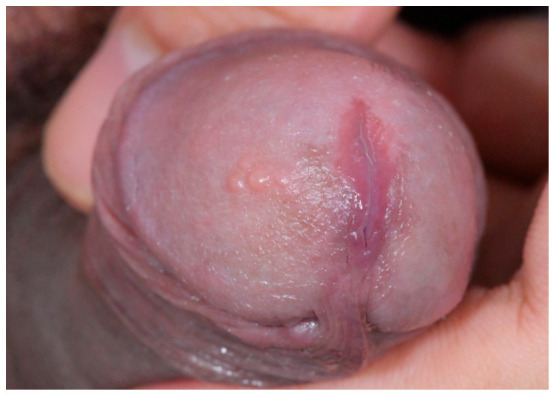
Three flesh-colored papules on the glans penis.

A healthy 23-year-old man presented with a 3-year history of gradually enlarging, mildly pruritic, flesh-colored papules on the distal glans of the penis. He denied a history of trauma or prior treatment. No dysuria, discharge, or erectile symptoms were observed. He denied any sensory alterations, including paraesthesia, hyperaesthesia, or numbness, in the glans penis. There was no personal or family history of melanoma or neurofibromatosis. On examination, three firm, dome-shaped papules (2–4 mm) were fixed to the overlying skin, yet mobile over deeper structures. No regional adenopathy, induration, or fluctuation was observed (Figure 1). Dermoscopy findings revealed a non-specific whitish structureless area.

**Figure 2:**
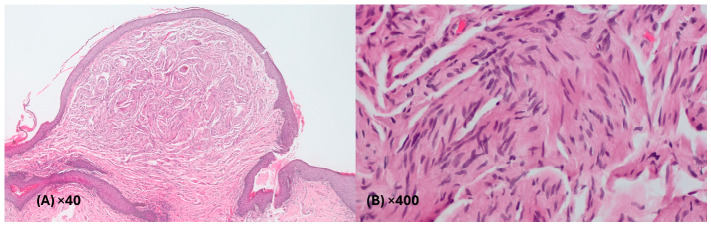
(**A**,**B**), Histopathological imaging revealed a well-circumscribed dermal nodule partially encapsulated by fibrous connective tissue. Within the tumor, proliferation of spindle-shaped cells formed an interlacing fascicle with a small palisade pattern, and prominent clefts delineated these fascicles. (Hematoxylin-eosin staining (H&E), original magnification ×40, 400).

**Figure 3:**
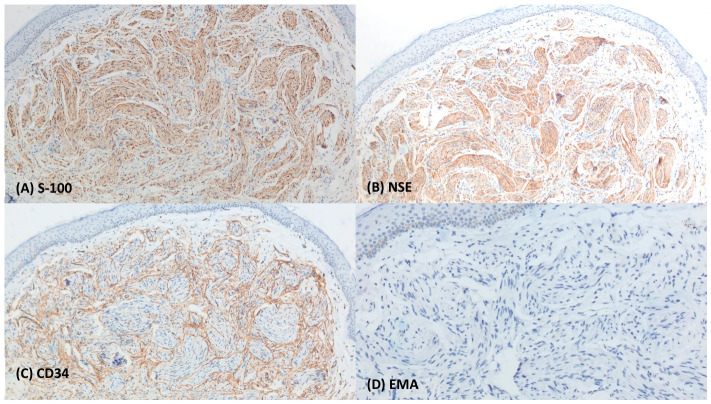
(**A**) S-100 protein positivity in tumor cells indicated that these cells were Schwann cells. (**B**) NSE staining highlighted the presence of numerous axons within the tumor tissue. (**C**) CD34-positive staining of endoneural cells inside the peripheral nerve structure. (**D**) EMA staining of the tumor capsule, which was negative in our case.

Histopathology revealed a well-circumscribed, encapsulated dermal tumor of spindle cells arranged in short fascicles with focal palisading and artificial clefts. Tumor cells had eosinophilic cytoplasm and oval to wavy basophilic nuclei, without pleomorphism or mitoses. The epidermis was normal. (Figure 2A,B). Immunohistochemistry revealed S-100 positivity and EMA (epithelial membrane antigen) negativity, indicating Schwann cell differentiation. The capsule featured flattened cells with a perineurial phenotype, though EMA was negative. CD34-positive endoneural cells were present at the tumor periphery. Scattered intralesional axons stained with Neuron-Specific Enolase (NSE) were interspersed among S-100-positive cells (Figure 3A–D).

## 2. What Is the Diagnosis?

A. Pearly penile papules

B. Lymphangioma

C. Palisaded encapsulated neuroma

D. Schwannoma

E. Condyloma acuminata

## 3. Diagnosis

C. Palisaded encapsulated neuroma.

## 4. Discussion

Palisaded encapsulated neuroma (PEN) is a benign dermal nerve sheath tumor accounting for 8–25% of cutaneous neuromas [1]. PEN is characterized by the hyperplastic proliferation of typical components of peripheral nerve fibers, with no known tendency for malignant transformation [2]. Its pathogenesis is unclear, although repetitive microtrauma to small cutaneous nerves has been proposed.

PEN is clinically characterized as a solitary, painless, firm papule or nodule, 2–6 mm, most often located on the face and mucocutaneous junction; penile involvement is exceptionally rare. To the best of our knowledge, only five previously published cases of PEN arising specifically on the glans penis have been reported in the literature. It is usually observed in middle-aged adults between the ages of 40–60 years, with an equal sex distribution [2,3]. The presentation of this lesion requires differential diagnosis to distinguish it from other conditions such as pearly penile papule, lymphangioma, condyloma acuminata, intradermal nevus, epidermal cyst, basal cell carcinoma, and adnexal tumors, particularly schwannoma.

Clinically, the lesion resembled a small milium-like whitish papule. On dermoscopy, however, the lesion showed only non-specific whitish structureless areas, without a disease-specific pattern or diagnostically useful vascular structures. Although previous reports of palisaded encapsulated neuroma/solitary circumscribed neuroma have described homogeneous white-to-ivory areas and, in some cases, arborizing vessels, these features were not clearly appreciable in the present lesion. Therefore, dermoscopy did not materially aid the preoperative diagnosis, which was ultimately established on histopathological and immunohistochemical grounds [4].

Histopathology of PEN is characterized by well-circumscribed intradermal nodules. They consist of a partially encapsulated aggregation of Schwann cells organized into interlacing fascicles separated by small clefts and interspersed with a variable quantity of minute axons. While the lobular growth pattern is most frequently observed, other morphological types including plexiform, multinodular, and fungating forms are noted [3,5].

The histological differential diagnosis includes schwannoma, solitary neurofibroma, traumatic neuroma, and leiomyoma. Distinguishing PEN from schwannoma is of particular importance. Schwannomas characteristically arise in the subcutaneous tissue or deeper soft tissue and exhibit distinct Antoni A areas (compact spindle cells with nuclear palisading forming Verocay bodies) and Antoni B areas (loosely arranged hypocellular zones with myxoid stroma). Critically, schwannomas lack intratumoral axons, whereas the identification of interspersed axons on NSE or neurofilament protein (NFP) staining is a defining feature of PEN that strongly supports its diagnosis over schwannoma. In the present case, the absence of Antoni A/B areas and Verocay bodies, combined with NSE-highlighted intralesional axons, effectively excludes schwannoma.

Solitary neurofibroma is another important differential diagnosis. Neurofibromas are unencapsulated lesions with diffuse infiltrative growth, mucopolysaccharide-rich stroma, a heterogeneous cell population including Schwann cells, perineurial cells, and fibroblasts, and diffuse CD34 stromal positivity throughout the lesion. In contrast, the present case demonstrates a circumscribed and partially encapsulated architecture, a fascicular growth pattern with characteristic cleft formation, and CD34 staining that highlights endoneurial cells at the periphery of the lesion rather than diffuse stromal positivity—a pattern more consistent with PEN than with solitary neurofibroma. Furthermore, the presence of intratumoral axons and the absence of GFAP expression provide additional support for PEN over neurofibroma. Traumatic neuromas exhibit haphazard axonal proliferation within a fibrotic stroma and lack encapsulation. Leiomyomas demonstrate desmin-positive smooth muscle bundles and are readily distinguished by immunohistochemistry [1,3].

The immunohistochemical antigens employed to distinguish PEN from other cutaneous neoplasms include S-100, EMA, neurofilament, CD34, collagen type 4, factor XIIIa, and GFAP. Immunostaining of PEN reveals positivity for S100, vimentin, CD34, collagen type 4, factor XIIIa, and NSE, suggesting Schwann cell differentiation of spindle cells and positivity for EMA at the perineural capsule, whereas GFAP is absent. Schwannoma is characterized by the presence of S-100, collagen type 4, and factor XIIIa, but lacks EMA, neurofilament, CD34, and GFAP. Neurofibroma exhibits positive expression for S-100 and GFAP, with variable or weak expression for EMA, neurofilament, CD34, and collagen type 4, and is negative for Factor XIIIa. Traumatic neuroma is positive for S-100 and GFAP, with variable expression for EMA, neurofilament, CD34, collagen type 4, and Factor XIIIa [6].

The immunohistochemical pattern observed in our case aligns with the findings of other studies, which have consistently demonstrated S-100-positive staining in Schwann cells. NSE staining highlighted numerous axons interspersed throughout the tumor tissue. CD34 highlights endoneurial cells within the peripheral nerves. However, in our case, staining for epithelial membrane antigen (EMA) and collagen type 4 was negative. It is important to distinguish between the histological impression of partial encapsulation on haematoxylin and eosin (H&E) staining and immunohistochemical evidence of a continuous perineurial capsule. While the lesion appeared circumscribed with a fibrous investment on H&E, the negative EMA result indicates the absence of a convincing immunohistochemically confirmed perineurial sheath. Characteristically, PEN is surrounded by a perineurial sheath highlighted by EMA; however, the integrity of this capsule is frequently compromised, with focal-to-extensive loss reported in 73% of cases [1,7]. This disruption likely explains the absence of EMA staining in the perineurial cells in our case.

Regarding the demonstration of intratumoral axons in our case, Neuron-Specific Enolase (NSE) was utilized as a surrogate marker to confirm the neural origin of the spindle cell proliferation, given its institutional availability. Compared with neurofilament protein (NFP), NSE offers greater sensitivity—particularly in immature or poorly differentiated cells—and more consistent and intense immunoreactivity across neural and neuroendocrine tumors; however, it is less specific, as it may also label non-neural cells and neuroendocrine elements beyond the axonal compartment. While NFP remains the preferred marker for definitive axonal confirmation, the characteristic histomorphological features on haematoxylin and eosin (H&E) staining—a well-circumscribed dermal nodule composed of Schwann cell aggregations arranged in interlacing fascicles, separated by cleft-like artifacts and interspersed with delicate axons—remain highly characteristic of PEN and support the diagnosis in the appropriate clinical context [8].

Simple complete excision of these lesions is both diagnostic and curative. Recurrence and malignant transformation have not yet been reported. Additional systemic disease workup is unnecessary because PEN lacks associations with neurocutaneous syndromes. Ablative lasers are anecdotally effective for cosmetically sensitive areas. Our patient was referred for complete surgical excision [9].

Herein, we report a rare case of PEN involving the glans penis and highlight that combined histopathology and immunohistochemistry can facilitate the differentiation of PEN from other persistent glandular lesions [10,11,12,13,14].

## 5. Conclusions

We report a rare case of palisaded encapsulated neuroma (PEN) arising on the glans penis, an exceptionally uncommon site for this benign peripheral nerve sheath tumor. Diagnosis was established on classic H&E histopathological features, supported by S-100 positivity, NSE-highlighted intralesional axons, CD34-positive endoneurial cells, and negative EMA—consistent with PEN exhibiting incomplete encapsulation. This case highlights that PEN should be considered in the differential diagnosis of persistent flesh-colored penile papules, where integrated clinicopathological and immunohistochemical evaluation is essential for accurate diagnosis. Complete surgical excision remains the treatment of choice and is curative.

## Data Availability

The data presented in this study are available on request from the corresponding author. The data are not publicly available due to patient privacy considerations.
